# Effect of curcumin on γ–ray-induced cell response

**DOI:** 10.1093/jrr/rrac101

**Published:** 2023-01-11

**Authors:** Nora Kostova, Albena Staynova, Ljubomira Popova-Hadjiiska, Dimka Georgieva, Ilonka Ivanova, Nevena Aneva, Margarita Atanasova, Rositsa Hristova

**Affiliations:** Department of Radiobiology, National Centre of Radiobiology and Radiation Protection, “Sv. Georgi Sofiiski” Str., No 3; Sofia, 1606, Bulgaria; Department of Radiobiology, National Centre of Radiobiology and Radiation Protection, “Sv. Georgi Sofiiski” Str., No 3; Sofia, 1606, Bulgaria; Department of Radiobiology, National Centre of Radiobiology and Radiation Protection, “Sv. Georgi Sofiiski” Str., No 3; Sofia, 1606, Bulgaria; Department of Radiobiology, National Centre of Radiobiology and Radiation Protection, “Sv. Georgi Sofiiski” Str., No 3; Sofia, 1606, Bulgaria; Department of Radiobiology, National Centre of Radiobiology and Radiation Protection, “Sv. Georgi Sofiiski” Str., No 3; Sofia, 1606, Bulgaria; Department of Radiobiology, National Centre of Radiobiology and Radiation Protection, “Sv. Georgi Sofiiski” Str., No 3; Sofia, 1606, Bulgaria; Department of Radiobiology, National Centre of Radiobiology and Radiation Protection, “Sv. Georgi Sofiiski” Str., No 3; Sofia, 1606, Bulgaria; Department of Radiobiology, National Centre of Radiobiology and Radiation Protection, “Sv. Georgi Sofiiski” Str., No 3; Sofia, 1606, Bulgaria

**Keywords:** curcumin, radioprotective effect, γ-rays, γ-H2AX/53BP1 foci, FISH analysis of translocations

## Abstract

The purpose of the present study is to evaluate the effect of curcumin as a natural compound against radiation induced γ-foci and stable chromosome aberrations. Whole blood samples form three human volunteers were pretreated with curcumin at different concentrations (0.5, 10, 20 and 100 μg/ml). After 1-hour incubation, the lymphocytes were exposed to γ-rays (0.05, 0.5, 1 and 2 Gy). Radiation induced changes in cells were quantified using γ-H2AX/53BP1 assay and FISH analysis. Our results have shown that curcumin significantly reduced the frequency of both γ-foci and translocations. We found concentration-dependent increase of curcumin protective effect on γ-H2AX/53BP1 foci formation at all radiation doses. Concerning the translocations, after 0.05 and 0.5 Gy γ-rays the values of genomic frequencies are comparable within each dose and we did not observe any impact of curcumin. The most protective effect after 1 Gy exposure was found at 100 μg/ml curcumin. At 2 Gy irradiation, the maximum protection was achieved at 0.5 and 10 μg/ml of curcumin. Concentrations of 20 and 100 μg/ml also prevent lymphocytes but to less extent. Our *in vitro* study indicates radioprotective efficacy of curcumin against γ-ray induced damages in human lymphocytes. This observation suggests that curcumin may play a role to protect patients undergoing radiological procedures.

## INTRODUCTION

The living cells are highly sensitive to ionizing radiation (IR). The most crucial lesions as a consequence of IR are DNA double strand breaks (DSBs), arising linearly with radiation dose [1]. Once appeared, they trigger a number of signal pathways, particularly cellular repair response. The earliest step in this process is phosphorylation of nucleosome core histone H2AX in the vicinity of DSBs [2]. The phosphorylated form, termed γ-H2AX, accumulates at the sites of damaged DNA in discrete nuclear spots ‘foci’, that are visible after immunostaining. Gamma-H2AX foci appear rapidly, 1 to 3 minutes after irradiation, reach their peak at 30 min and decrease to background level within several hours or days depending on dose [3]. Immunohistochemical localization of γ-H2AX is used for assessment of DNA damage and DNA repair as well [2]. Another protein, tumor suppressor p53 binding protein 1 (53BP1) has been also reported co-localized to DSBs [4]. It is believed that γ-H2AX and 53BP1 both together provide a scaffold structure for DSBs repair, recruiting other reparative proteins [5]. On the other hand, misrepaired DSBs induce structural chromosomal aberrations like dicentrics, rings, translocations and fragments. All these aberrations increase in dose dependent manner [6]. They could result in either loss or rearrangement of genetic information, cell death or malignant transformation [7].

Regardless of its deleterious effect, IR is widely used in medicine for both diagnostic and treatment. About 80% of cancer patients need radiotherapy, which is a highly effective tool to kill tumor cells, but also affects the surrounding normal cells. For optimal therapeutic results, balance between irradiation dose and normal tissue protection is required. In this line, the scientific interest is focused on agents that could prevent or mitigate radiation induced damage in healthy tissue. A variety of synthetic radioprotectors has been known, but most of them are high cost, toxic or have side effects [8].

Curcumin, a pigment extracted from turmeric plant *Curcuma longa* has been widely investigated over the years, because of its biological and pharmacological effects. The molecule of this unique natural compound contains three reactive functional sites (two phenolic groups and one β-diketone moiety), which are related to its high antioxidant activity and ability to neutralize the free active oxygen radicals resulting from various mutagenic factors, particularly IR [9]. Furthermore, curcumin possess anti-inflammatory, anti-tumor, pro- and anti-apoptotic effects [10–12]. Its anti-proliferative efficacy has been reported in leukaemia, colon and breast cancer [13–15]. It is also implicated in gene expression regulation of various transcription factors, growth factors and their receptors, nuclear factors, hormones [9]. According to the literature, curcumin has a dual mode of action after irradiation [16]. It possesses both radioprotective and radiosensitizing activities, although the mechanisms underlying such properties are still unclear.

Most of investigations concerning the role of curcumin as radioprotector respectively as antimutagen, are mainly *in vivo* on animals [17, 18]. The *in vitro* studies in this field are controversial. Thus, curcumin has been reported to decrease the number of radiation induced DSBs or γ-H2AX foci [19, 20]. Similar effect was found on both micronuclei and dicentrics whose frequency was reduced after pre-treatment with different curcumin concentrations before γ-ray exposure [21, 22]. Contradictory, some authors discovered so-called clastogenic effect which leads to DSBs accumulation or increased chromosome aberrations frequency resulted from different curcumin concentrations used [23, 24]. Obviously, expression of either antimutagenic or clastogenic efficacy could depend on experimental conditions. To shed more light on this area, we use a larger range in respect to both curcumin concentration and γ-rays dose. Furthermore, up to our knowledge all studies till now concern curcumin effect on unstable chromosomal aberrations (micronuclei and dicenrtics), which are eliminated during the next cell division cycle. The aim of the present study is to elucidate how curcumin affect stable aberrations, such as translocations, that could be observed many years post irradiation and are related to cancer risk. As the translocations are due to misrepaired DSBs, we have also investigated curcumin effect on early DNA damage stages after γ-ray exposure, when γ-H2AX foci appear.

## MATERIALS AND METHODS

Stock solution of curcumin (Sigma-Aldrich) using 96% ethanol as a solvent was prepared the day before irradiation and stored at −20°C in darkness.

### Blood collection and irradiation conditions

Whole blood samples from three healthy donors (two females and one male, aged between 34 and 45 years) were taken with informed consent and the approval of a local ethics committee (Ref. No. 0001/15.02.2019). The blood samples were collected in sterile EDTA or lithium heparin tubes depending on the method used (γ-H2AX/53BP1 assay or FISH respectively). Curcumin was added one hour before irradiation at different concentrations (0.5; 10; 20 and 100 μg/ml). The samples were incubated for one hour at room temperature in dark. Thereafter they were exposed to ^60^Co γ-rays using various irradiation doses (0.05; 0.5; 1 and 2 Gy), at a dose rate of 0.246 Gy/min (PTW-30013 ionization chamber, PTW). Both curcumin non-treated and non-irradiated samples served as controls. To exclude ethanol toxic effect on investigated biomarkers, non-irradiated cells treated with ethanol (experimental concentration below 0.1%) were also analyzed. We have not found any clastogenic effect of ethanol.

### γ-H2AX/53BP1 immunofluorescence staining

Thirty minutes after *in vitro* irradiation peripheral blood mononuclear cells were isolated using Histopaque 1077 (Sigma-Aldrich), according to the manufacturer’s protocol. The cells were washed three times in phosphate-buffered saline (PBS), resuspended in PBS and counted automatically (Countess™, Invitrogen). Their viability was >85%, estimated by trypan blue dye exclusion assay. Thereafter they were spotted onto microscope slides (Thermo scientific) at a concentration of ~1x10^6^ cells/ml. Cells were then fixed in 2% formaldehyde (Polysciences Incorporated), permeabilized in 0.25% Triton X-100 (Sigma-Aldrich), blocked with 1% BSA/PBS (Sigma-Aldrich) and incubated with primary antibody (mouse anti-phospho-H2AX Ser139, Millipore, *Cat # 05-636-I*), diluted to 1:500 in 1% BSA/PBS. To verify that γ-H2AX foci really mark DSB, we performed double-staining with specific primary antibody versus other DSB marker (rabbit polyclonal to 53BP1, Abcam, *Cat # ab36823*), diluted to 1:400 in 1% BSA/PBS. After 45 min of incubation, the pellet was washed with 1% BSA/PBS and the secondary antibodies were added (Alexa Fluor 488 goat anti-mouse, Invitrogen, *Cat # A11029*) and (Alexa Fluor 555 goat anti- rabbit, Invitrogen, *Cat # A21429*) which were diluted to 1:500 in 1% BSA/PBS. After 30 min of incubation (the samples were stored in dark), the pellet was washed with 1% BSA/PBS. The slides were dried, mounted with DAPI Vectashield solution (Vector Laboratories) and stored at 20°C until analysis.

### Foci analysis

**Fig. 1:**
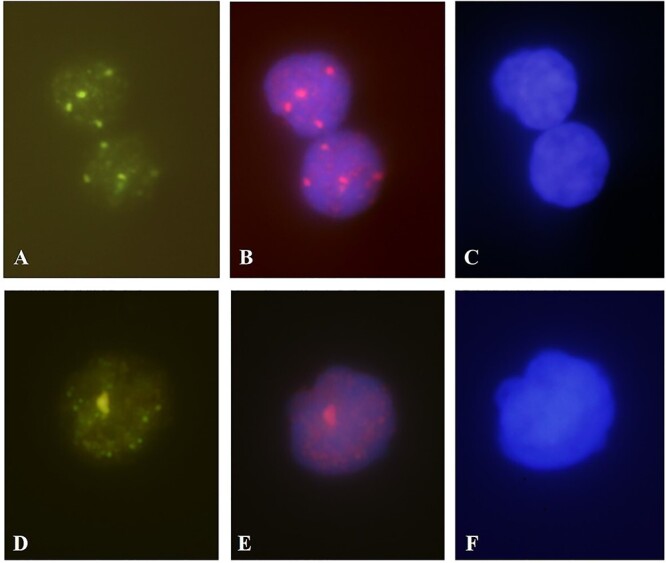
Immunolocalization of γ-H2AX/53BP1 foci in lymphocytes, irradiated with 0.5 Gy γ-rays (×100 objective). A, B and C – curcumin non-treated cells; D, E and F – cells treated with 100 μg/ml curcumin; Visualization of γ-H2AX protein (A) and (D); Visualization of 53BP1 protein (B) and (E); Nuclear DAPI staining (C) and (F).

Microscope slides were analyzed using an Olimpus BX41 fluorescence microscope (equipped with filter blocks for excitation of red, green and blue fluorescence). For each experimental point, between 100 and 200 cells from a donor were scored. All counting was performed by eye during the microscopic process using a 100 x immersion objective (Fig. 1). The yield of γ-H2AX/53BP1 foci number was estimated using the formula *Σ foci/n total cells*, where *Σ foci* shows the total number of foci in the scored cells and *n total cells* presents the total number of cells analyzed at that point. The background frequency of γ-H2AX foci was obtained from non-irradiated samples.

### Fluorescent in situ hybridization

#### Culture conditions

Whole blood samples treated as described above were cultured in RPMI 1640 medium supplemented with 10% fetal bovine serum, antibiotics (50 U/ml penicillin and 50 μg/ml streptomycin) (Sigma-Aldrich) and stimulated with 2% phytohemagglutinin (Gibco-Liftechnologies). The culturing of the cells was carried out at 37°C in 5% CO_2_ for 50 hours. After 48 h culture time cells were arrested in metaphase by demecolcine (0,1 μg/ml; Invitrogen). They were harvested, treated with a hypotonic solution and fixed routinely as described previously [25]. Metaphases were spread on slides and after drying stored at −20°C until use.

#### FISH staining

Chromosome spreads were pretreated with pepsin-HCL (10 mM) for 4 min at 37°C and immediately washed in PBS. The slides were then placed into a Coplin jar containing 50 mM MgCl_2_/PBS and fixed in 1% formaldehyde for 10 min. After that, they were subsequently dehydrated in 70%, 90% and 100% ethanol and air-dried.

Three-colored FISH was performed using XCyting DNA Probes (MetaSystems), according to manufacturer’s protocol. A cocktail of pre-mixed fluorochrome labeled probes was used: chromosome #1 (FITC), chromosome #4 (Texas red) and chromosome #11 (FITC/Texas red). At the last step, metaphase chromosomes were counterstained and mounted with DAPI (Sigma-Aldrich) in anti-fading solution Vectashield (Vector Laboratories). Hundreds of cells for each dose point and each curcumin concentration were scored (Table 2).

**Table 2:** Genomic frequency of AST in curcumin-treated/non-treated peripheral blood lymphocytes, after γ-irradiation (*P* < 0.05)

Dose	Curcumin	Genomic frequency of AST			Mean genomic	Total number of cells analyzed
(Gy)	(μg/ml)	Donor I	Donor II	Donor III	frequency ± SE	
	0	0.003	0.005	0.035	0.014 ± 0.002	5613
	0.5	0	0.011	0.016	0.009 ± 0.002	2809
0	10	0.006	0	0.011	0.006 ± 0.002	2529
	20	0.006	0.006	0.025	0.012 ± 0.002	3336
	100	0	0.007	0.008	0.005 ± 0.001	2723
	0	0	0.006	0.03	0.012 ± 0.002	3686
	0.5	0	0.011	0.018	0.01 ± 0.002	3187
0.05	10	0.003	0.008	0.02	0.01 ± 0.002	2468
	20	0.009	0.008	0.021	0.013 ± 0.002	2715
	100	0.005	0.004	0.019	0.009 ± 0.002	2307
	0	0.027	0.028	0.037	0.031 ± 0.003	3110
	0.5	0.027	0.034	0.043	0.035 ± 0.003	3482
0.5	10	0.019	0.026	0.043	0.029 ± 0.003	2578
	20	0.026	0.035	0.052	0.038 ± 0.004	2546
	100	0.034	0.04	0.039	0.038 ± 0.004	2757
	0	0.131	0.133	0.154	0.126 ± 0.007	3116
	0.5	0.106	0.104	0.098	0.103 ± 0.006	2753
1	10	0.076	0.092	0.099	0.089 ± 0.006	2784
	20	0.09	0.112	0.089	0.097 ± 0.006	2951
	100	0.09	0.095	0.073	0.067 ± 0.007	1684
	0	0.373	0.422	0.441	0.412 ± 0.013	2628
	0.5	0.208	0.284	0.274	0.255 ± 0.011	2168
2	10	0.253	0.308	0.223	0.261 ± 0.011	2069
	20	0.261	0.265	0.329	0.285 ± 0.011	2497
	100	0.238	0.313	0.331	0.294 ± 0.012	2152

AST – apparently simple translocations; SE – standard error

#### Chromosome aberration analysis

**Fig. 2:**
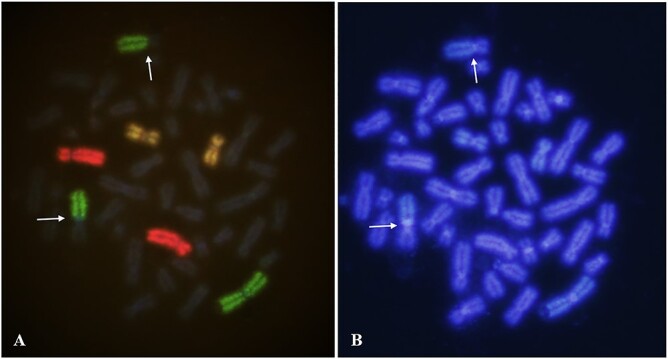
Three-colored FISH of chromosome pairs #1 (green), #4 (red) 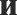 #11 (yellow) (×100 objective). The arrows show reciprocal translocation between chromosome #1 and unstained chromosome (A); The same metaphase counterstained with DAPI (B).

Only intact cells with ~46 chromosomes and all three painted chromosome pairs were scored. Classification of aberrations was carried out using PAINT nomenclature proposed by Tucker *et al.* [26]. Exchanges involving one painted and one non-painted chromosome were defined as simple. Apparently simple translocations (AST) were classified as reciprocal (Fig. 2) or terminal, but for the purpose of this study they were added together. Rearrangements with three or more breaks in two or more chromosomes were considered as complex.

Since FISH analysis of three-colored aberrations detect only a portion of total aberrations present in the cell, the exchange frequency was converted to a whole-genome equivalent according to IAEA manual (2011) [6]. The calculated factor (*Fp/Fg*) for chromosomes #1, #4, and #11 was either 0.338 for female or 0.343 for male. We have applied the following formula:


$$\begin{align*} Fp/ Fg=&2.05\left[\left({f}_1\left(1-{f}_1\right)+{f}_4\left(1-{f}_4\right)+{f}_{11}\left(1-{f}_{11}\right)\right)\right.\\ &\left.-\left({f}_1{f}_4+{f}_4{f}_{11}+{f}_1{f}_{11}\right)\right], \end{align*}$$

where *Fp* is the frequency of translocations detected by FISH, *f_p_* is the painted fraction of the genome, and *Fg* is the genomic aberration frequency.

FISH analysis was carried out manually by Leica DM 2000 LED microscope equipped with MC170HD digital camera.

Statistical analyses were performed by using statistical software (IBM SPSS 19 and MS Excel). The data were analyzed using one-way ANOVA analysis with post hoc tests. The differences were considered as significant when the *P*-value was < 0.05. Correlation was assessed using Spearman’s rank correlation coefficient.

## RESULTS

### γ-H2AX/53BP1 assay

**Fig. 3:**
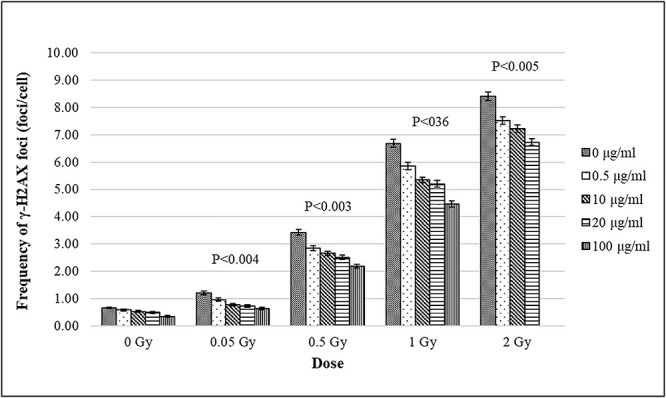
Effect of curcumin (concentrations: 0; 0.5; 10; 20 and 100 μg/ml) on the frequency of γ-foci, induced after *in vitro* irradiation with γ-rays (0; 0.05; 0.5; 1 and 2 Gy); (*P* < 0.05). Values are given as mean frequency; Error bars indicate standard error.

The mean frequency ± SE of γ-H2AX/53BP1 foci in non-treated lymphocytes from three donors was 0.65 ± 0.03 foci/cell (Table 1). In *in vitro* irradiated samples, γ-H2AX and 53BP1 foci were overlapping and co-localized. After radiation exposure (0.05; 0.5; 1 and 2 Gy), the mean yield of γ-H2AX/53BP1 foci in curcumin untreated lymphocytes showed dose-dependent increasing from 1.20 ± 0.06 to 8.41 ± 0.17 foci/cell. We observed negative correlation between curcumin concentration and γ-foci formation. Pretreatment with curcumin decreased the frequency of γ-foci gradually with increasing the tested concentration (0.5; 10; 20 and 100 μg/ml) at all radiation doses. After samples irradiation with 0.05 Gy, the frequency decreased in the range between 20% at 0.5 μg/ml and 47% at 100 μg/ml compared to control level without curcumin (Fig. 3). After irradiation with 0.5 Gy the frequency decreased in the range between 17% at 0.5 μg/ml and 36% at 100 μg/ml compared to control level without curcumin. At 1 Gy irradiation the frequency decreased in the range between 12% at 0.5 μg/ml and 33% at 100 μg/ml compared to control level. After 2 Gy γ-ray exposure the frequency was between 11% at 0.5 μg/ml and 16% at 20 μg/ml compared to no curcumin control level (we were not able to analyze the lymphocytes incubated with 100 μg/ml of curcumin and irradiated with 2 Gy because of bad signal quality). Our results demonstrate that pretreatment with curcumin before irradiation in a dose range of 0.05 to 2 Gy significantly reduced the frequency of γ-H2AX/53BP1 foci in peripheral blood lymphocytes (*P* < 0.05).

**Table 1:** Frequency of γ-H2AX/53BP1 foci ± SE in curcumin-treated/non-treated lymphocytes from three donors, before and after γ-irradiation (*P* < 0.05)

Curcumin concentration	Radiation dose (Gy)				
	0	0.05	0.5	1	2
0 μg/ml	0.65 ± 0.03	1.20 ± 0.06	3.43 ± 0.09	6.68 ± 0.15	8.41 ± 0.17
0.5 μg/ml	0.58 ± 0.03	0.96 ± 0.06	2.85 ± 0.10	5.85 ± 0.14	7.52 ± 0.14
10 μg/ml	0.53 ± 0.04	0.77 ± 0.05	2.66 ± 0.07	5.36 ± 0.10	7.22 ± 0.13
20 μg/ml	0.49 ± 0.04	0.73 ± 0.05	2.51 ± 0.08	5.21 ± 0.13	6.73 ± 0.13
100 μg/ml	0.35 ± 0.03	0.64 ± 0.05	2.18 ± 0.07	4.47 ± 0.12	^*^

^*^Data are not available; SE – standard error

### FISH

Curcumin effect on stable translocations was investigated *in vitro* by three-colored FISH. A total number of approximately 70 000 metaphase spreads were scored in FISH-stained preparations.

**Fig. 4:**
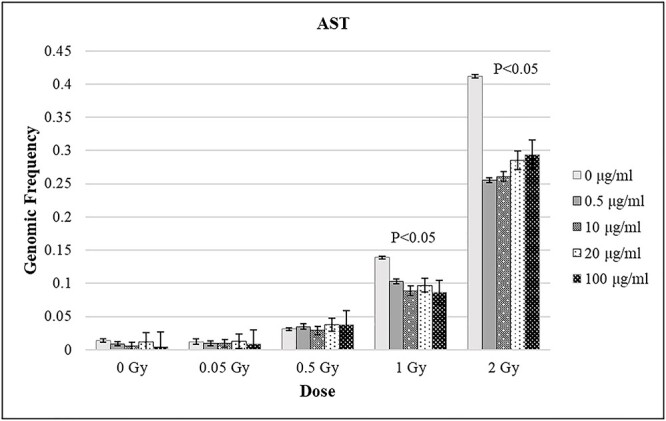
Curcumin effect on AST genomic frequency in peripheral blood lymphocytes, after γ-irradiation (ranged from 0 Gy to 2 Gy) (*P* < 0.05); Error bars indicate standard error.

The results from analysis are presented in Table 2. The mean AST genomic frequency ± SE from three volunteers showed dose depended increasing after *in vitro* irradiation, ranged from 0.014 ± 0.002 to 0.412 ± 0.013 in curcumin non-treated lymphocytes (Table 2, Fig. 4). Some differences between the donors due to individual variability were observed. Curcumin effect on chromosome translocations occurrence after γ-ray exposure was presented as the percentage decrease of their genomic frequency from the respective initial value. The level of radioprotection for each concentration of curcumin and each dose was estimated, using the following formula:


$$Y=100-\frac{Genomic\ Frequency\ with\ curcumin\ {}^\ast 100}{Genomic\ Frequency\ with out\ curcumin}$$

When the lymphocytes were pretreated with curcumin and irradiated with lower doses of γ-rays (0.05 Gy or 0.5 Gy), no curcumin effect on chromosomal aberrations appearing was found (Table 2; Fig. 4). In contrast, at 1 Gy irradiation all curcumin concentrations lead to significantly reduced translocation frequencies (*P* < 0.05). The mean levels of radioprotection vary from 25.9% at 0.5 μg/ml of curcumin to 38.1% at 100 μg/ml of curcumin (Fig. 4). When the lymphocytes were exposed to 2 Gy γ-rays, the maximum level of radioprotection was again 38.1%, but in this case the most protective curcumin concentration was 0.5 μg/ml. Similarly, all curcumin concentrations significantly reduced mean translocation frequencies ranged from 28.6% to 38.1% (*P* < 0.05).

Curcumin also affects complex translocations occurrence when lymphocytes were exposed to 2 Gy γ-rays. Data are shown in Table 3. We found that pre-treatment with 100 μg/ml curcumin reduced the percent of complex aberrations 2-fold, compared to curcumin non-treated cells.

**Table 3:** Curcumin effect on complex aberrations after 2 Gy *in vitro* irradiation

Curcumin	0 μg/ml	0.5 μg/ml	10 μg/ml	20 μg/ml	100 μg/ml
Total number of cells analyzed	2628	2168	2069	2497	2152
Total number of complex aberrations	31	24	16	24	13
Frequency of complex aberrations	0.012	0.011	0.008	0.01	0.006

## DISCUSSION

IR can interact directly with DNA or through generation of reactive oxygen species (ROS) which in turn are capable of damaging DNA [27]. Induction of DNA DSBs is one of the most critical effects occurred after irradiation. This kind of lesions could cause genomic instability, cell death or can increase risk of malignant transformation [28]. Phosphorylation of many histone H2AX molecules in the vicinity of a DSB generates so-called γ-H2AX and is a very important step in initiation of DNA damage response. The creation of fluorescent antibody specific for γ-H2AX allowed to the development of a reliable assay that make it possible to detect the γ-foci formed at the sites of DSBs.

In the present study, we have tested the effect of four different curcumin concentrations on both γ-H2AX/53BP1 foci and translocations frequency induced after γ-ray exposure. Curcumin treatment of human whole blood from three donors before irradiation significantly decreased γ-foci frequency compared to irradiated samples without curcumin (*P* < 0.05). The lowest reduction was by 11% found in lymphocytes incubated with 0.5 μg/ml of curcumin and irradiated with 2 Gy γ-rays. The highest reduction was by 47% observed in lymphocytes incubated with 100 μg/ml of curcumin and irradiated with 0.05 Gy. The decreased levels of radiation induced γ-H2AX/53BP1 foci showed that curcumin could prevent DNA from DSBs due to its ROS scavenging activity. We observed that the reduction in γ-foci frequency within each dose is dependent on curcumin concentration (Fig. 3). On the other hand, the frequency of γ-H2AX/53BP1 foci is getting higher with the dose for each tested concentration. This is probably due to the fact that with increasing the radiation dose, the number of free radicals also increases, and in turn, the actual curcumin concentration is insufficient to quench the produced radicals.

To our knowledge, there are few studies related to the effect of different compounds on radiation induced γ-H2AX/53BP1 foci. Recently, Kuefner *et al.* investigated the effect of a mix of antioxidants (vitamin E, vitamin C, carotenoids, etc.) on the γ-H2AX foci appearance in X-ray irradiated human lymphocytes. They have detected a significant reduction in γ-H2AX foci, when the cells were pre-treated with antioxidants for 60 minutes followed by 10 mGy X-ray irradiation [29]. Brand *et al.* concluded that pretreatment of blood samples with different antioxidants (vitamin E, vitamin C, N-acetyl-L-cysteine, etc.) prior to irradiation led to a significant reduction of γ-H2AX-foci [30]. It is known that curcumin has a strong capacity for scavenging a variety of ROS [31]. Being a hydrophobic molecule, it can pass through the cell membrane into the cytosol and thus can directly scavenge the free radicals [9, 32, 33]. It should be also pointed out that curcumin induces the activity of both the enzymatic and non-enzymatic antioxidants and it is another way to reduce levels of ROS and DSBs respectively [34]. Our results provide evidence of protective effect of curcumin against radiation induced DNA DSBs.

It is well known that translocations arise as a result of misrepaired DSBs [35]. As expected, we found radiation induced ASTs to follow linear-quadratic model described in the literature [36]. In the present study we observed that curcumin has no effect on chromosomal aberrations in lymphocytes irradiated with doses of either 0.05 Gy or 0.5 Gy. The mean genomic frequency of AST is not affected by pretreatment with different curcumin concentrations and values within each dose are comparable. It could be a result of cells capacity to restore DNA damages at the above mentioned doses. Principally, lower doses exposure induces less DSBs [1, 37]. Thus, our findings are in accordance with widely accepted view that 80% of DSBs are repairing during the first few hours post-exposure [35, 38, 39]. In the experimental system we used, lymphocytes were cultured for 50 hours before metaphase spreading while the repairing process was ongoing.

On the other hand, when curcumin non-treated lymphocytes were exposed *in vitro* to either 1 Gy or 2 Gy γ-rays, AST genomic frequency increased respectively 9-fold and 29-fold compared to its control level at 0 Gy. Obviously, in this case there are a lot of misrepaired DSBs. Our results concerning stable translocations showed curcumin protective effect after 1 Gy and 2 Gy γ-irradiation. We observed significant decline in ASTs frequency for all tested curcumin concentrations. According to complex aberrations induced after 2 Gy irradiation, pre-treatment with different curcumin concentrations leads to reduction in their frequency. All these findings are in agreement with previous *in vivo* and *in vitro* investigations. For example, *in vivo* experiments with mouse bone marrow have demonstrated significantly reduced micronucleus frequency after oral administration of curcumin followed by whole body exposure to gamma-rays [18]. Tawfik *et al.* have also found curcumin radioprotective activity on chromosomal aberrations in mice [17]. Similar effect has been reported in human lymphocytes after *in vitro* irradiation [21, 22]. In our study, the protective efficacy against 1 Gy exposure showed tendency towards concentration dependence, with a maximum protection at 100 μg/ml of curcumin (38.1%). Instead, slightly negative correlation between drug concentration and protective effect was found after 2 Gy exposure. The maximum protection was achieved at 0.5 μg/ml and 10 μg/ml curcumin, respectively 38.1% and 36.7%. The higher curcumin concentrations also protect lymphocytes but to less extent. Genomic frequencies of translocations were reduced by 30.8% or 28.6% compared to curcumin non-treated lymphocytes. It is difficult to explain the observed negative correlation but it should be taken in mind that curcumin has been supposed to exert its activity through various pathways [40]. Our results in respect to ASTs are in accordance with those obtained for dicentrics [22] or micronuclei [16]. Contradictory, some authors discovered increasing number of chromosomal aberrations in curcumin-treated Chinese hamster CHO cells followed by 2.5 Gy γ-irradiation [24]. These discrepancies could be due to different experimental conditions used (e.g. model systems; curcumin treatment at different phases of the cell cycle, etc.). At the same time, we did not find any clastogenic effect for all curcumin concentrations tested. However, Sebastia *et al.* supposed cytotoxic effect in lymphocytes exposed to 2 Gy γ-rays, when curcumin was applied in concentration of 500 μg/ml [22]. Both cytotoxic and genotoxic effect has been also found in non-irradiated tumor cell lines cells [41]. Further, investigations of Schwarz *et al.* have shown lower viability, higher cell death and potential apoptosis induction after both curcumin treatment and irradiation in pancreatic cancer cell lines [42]. These authors have assumed that curcumin could induce cell cycle arrest in the radiosensitive G2/M phase and thus radiosensibilizing the cells. Concerning our experiments, peripheral blood lymphocytes are not dividing cells, and curcumin may not have the same effect. Alternatively, it exerts radioprotective activity in healthy cells.

To have a better insight into the underlying protective effect of curcumin on human lymphocytes after irradiation, we have combined two methods: γ-H2AX foci assay and FISH analysis. While the first method is used to detect the induced DSBs soon after IR exposure, the second one identifies DNA damages persisting for at least one mitotic cycle or longer. The preliminary results from one healthy donor, published previously demonstrated protective effect of curcumin against γ-rays induced DSBs [43]. To get some more indications about interindividual variability, our investigation concerning γ-H2AX/53BP1 yield and AST genomic frequency was continued. The data presented in the current study have shown concentration dependent protective effect of natural compound after 1 Gy irradiation for both DSBs and ASTs. In this case we found a simple correlation: less γ-H2AX/53BP1 foci, less translocations. Not the same correlation was found, when cells were exposed to 2 Gy γ-rays. Similar, γ-foci decreased with increasing of curcumin concentrations. The lower frequency of γ-H2AX/53BP1 foci could be due to curcumin antioxidant activity and its ability to scavenge IR induced ROS. The higher compound concentration the more ROS are quenched. However, curcumin has been also reported to target DNA repair pathways [40]. Misrepaired DNA damages are realized physically through the translocations. The lowered γ-H2AX foci do not automatically mean lower translocations appearing. Although the found concentration-dependent reduction of γ-foci is clear, the mechanism of curcumin radioprotective activity on ASTs frequency remains still unclear. Further investigations are needed to clarify how this compound modulate the cell response after radiation exposure.

In conclusion, our results have shown that curcumin possess protective efficacy in all tested concentrations. We supposed two modes of action on irradiated cells for this compound: antioxidant activity and signaling pathways modulation. On the other hand, we did not find any clastogenic effect of curcumin even at concentration of 100 μg/ml. Along with observed radioprotective potential it could be a useful agent to prevent normal tissues damage and avoid adverse side effects of cancer therapy. Administration of curcumin together with traditional radiotherapy could improve the cancer patient clinical outcome.

## CONFLICT OF INTEREST

The authors declared no potential conflict of interest with respect to this study.

## FUNDING

This work was supported by Bulgarian National Science Fund; grant № KP-06-Н23/3/2018, funded by the Bulgarian government (Ministry of Education and Science).
